# Automated Image Sampling and Classification Can Be Used to Explore Perceived Naturalness of Urban Spaces

**DOI:** 10.1371/journal.pone.0169357

**Published:** 2017-01-04

**Authors:** Roger Hyam

**Affiliations:** Royal Botanic Garden Edinburgh, Edinburgh, United Kingdom; University of Melbourne, AUSTRALIA

## Abstract

The psychological restorative effects of exposure to nature are well established and extend to just viewing of images of nature. A previous study has shown that Perceived Naturalness (PN) of images correlates with their restorative value. This study tests whether it is possible to detect degree of PN of images using an image classifier. It takes images that have been scored by humans for PN (including a subset that have been assessed for restorative value) and passes them through the Google Vision API image classification service. The resulting labels are assigned to broad semantic classes to create a Calculated Semantic Naturalness (CSN) metric for each image. It was found that CSN correlates with PN. CSN was then calculated for a geospatial sampling of Google Street View images across the city of Edinburgh. CSN was found to correlate with PN in this sample also indicating the technique may be useful in large scale studies. Because CSN correlates with PN which correlates with restorativeness it is suggested that CSN or a similar measure may be useful in automatically detecting restorative images and locations. In an exploratory aside CSN was not found to correlate with an indicator of socioeconomic deprivation.

## Introduction

There is good empirical evidence that exposure to nature is beneficial for dealing with psycho-physiological effects of stress[1]. Just viewing images of nature has been shown to have a restorative effect [2–4]. The two main theoretical models to explain this are Stress Recovery Theory (SRT) [5] and Attention Restoration Theory (ART) [6]. These theories are complementary. SRT is more concerned with physiological and negative affect whilst ART is concerned with attentional fatigue [1]. A major question that remains to be answered is: What is it about natural environments that produces these benefits? [4].

Berman *et al* [4] used low level image characteristics (density of contrast changes, straight lines, colour saturation, hue diversity) to examine the underlying mechanisms that produce these benefits and found they predicted Perceived Naturalness (PN) with some accuracy.

PN was the average of scores given to an image by participants using a seven point Likert scale ranging from 1 ‘very manmade’ to 7 ‘very natural’. (PN should not be confused with perceived restorativeness as measured by PRS-11[7]).

However humans are also likely to interpret scenes within a semantic context even if they are influenced by low level patterns they see. Lin *et al* [8] demonstrated that drawing attention to street trees in images increased restorativeness value of those images suggesting that cognition has a role to play. Even in studies where awareness isn’t the main subject participants have their attention drawn to the natural aspects of the scenes as part of the procedure [3].

This study takes a complementary approach to Berman *et al* [4] by examining whether the high level semantics of objects found in an image have a bearing on PN and therefore possibly on restorative value. If restorativeness is mediated by meaning there is a role for education and training in enhancing the beneficial effects of urban green space. If restorativeness is not mediated by meaning then increasing the benefits the population gain from green space is a matter of landscape design alone. It is recognised that “The landscape is more than the enumeration of the things in the scene.” [9]. This study doesn’t look at the arrangement of objects but exploring how we might objectively enumerate them is a first step.

Two experiments were carried out. The first took the data and images used by Berman *et al [4]* and passed them through an automated image classification algorithm to test whether it could predict the PN of the images. The second experiment tested whether this approach could be applied to automatically sampled images from Google Street View and still predict PN.

The experiments make use of two web services provided by Google.

In February 2016 Google launched an image classification service called the Google Vision API[10]. This enables software developers to access image classification algorithms that have been built using very large training sets. Although the API (Application Programming Interface) provides a number of features, including face detection and optical character recognition, this study makes use of only the label detection feature. In response to the submission of an image it returns a list of labels for subjects the algorithm has detected along with a confidence score for each label. Examples of labels are “retail store”, “runner bean”, “sky” and “sports car”. The size of the total pool of labels isn’t published although likely to be in the thousands (this study encountered 430) and is probably dynamic. Image classification is an established field of machine learning. Although a single commercial API is used here, the principles explored are generic to image classification services and would apply to those from other providers as well as specifically trained classification algorithms.

Google Street View[11] was launched in 2007 and has been rolled out to many cities around the world. It consists of a series of photographic panoramas taken at precise points along streets and paths. These panoramas are integrated with maps and mobile phone applications including virtual reality viewers. The vast majority of panoramas are captured by a device containing a constellation of cameras mounted on the top of a normal saloon car although some have been captured by the device carried on a tricycle or backpack. The Google Street View Image API[12] allows software developers to extract rectilinear images from the panoramas. They specify a panorama by GPS coordinates or identifier, the direction to point the virtual camera and the angle of view (zoom level). A JPEG image is returned.

### Research Objectives

1. Can we predict the PN of images by listing their contents using automated image classification techniques? This is addressed by experiment 1.

2. Could this technique be applied to randomly sampled images in a city scale size study. This is addressed by experiment 2.

## Experiment 1: Semantic Assessment of Naturalness

### Materials

Berman *et al* [4] assessed PN for a set of images made up of three subsets. The high-natural set (n = 50 images) and low-natural (n = 50 images) had been used in a previous study [3] where participants had their attention and mood assesses prior to and following viewing the images. These images are therefore known to demonstrated a restorative effect. The third subset of images (n = 207 images) was of mixed urban green spaces had been scored for PN but not restorative effect. This experiment takes these 307 Berman *et al*’s [4] images and the PN scoring as a starting point because of their known provenance.

### Method

The 307 test images were passed to the Google Vision API and the labels returned for each image stored in an SQL database. Each label was then assessed (independently of associated images) by the author and placed into one of three broad semantic classes: natural, artificial, ambiguous. A Calculated Semantic Naturalness (CSN) score was derived for each image as the number of natural labels divided by the total labels for that image minus the number of artificial labels divided by the total labels for that image. This gives a scale from -1, most artificial, to +1 most natural.

$$\mathrm{CSN}=\frac{\mathrm{Natural Labels for Image}}{\mathrm{Total Labels for Image}}-\frac{\mathrm{Artificial Labels for Image}}{\mathrm{Total Labels for Image}}$$

### Results

A total of 293 labels were returned. On average each image was given 8.17 (max 18, min 1, sd 3.55) labels. Each label was returned for an average of 8.55 (max 105, min 1, sd 14.75) images. 140 labels were assigned to “natural”, 120 to the class artificial and 33 to ambiguous (Table 1).

**Fig 1:**
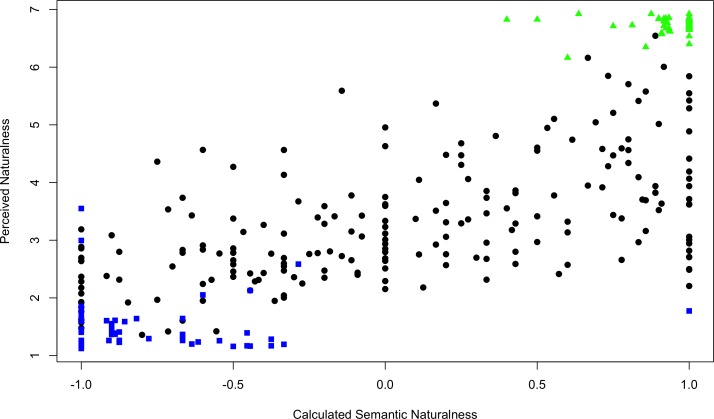
Correlation of Berman and Calculated Semantic Naturalness (Experiment 1).

Fig 1 is a plot of the PN from Berman et al against the CSN produced here. The high-natural (green triangles) and low-natural (blue squares) are clearly separated by CSN with a single low-natural outlier. The Pearson's product-moment correlation of PN to CSN is 0.7644 (p<0.01) 95% confidence between 0.7125 and 0.8072—a moderate to strong correlation. Minor changes to the assignment of labels had little impact on overall results correlations obtained.

**Table 1:** Number of labels returned.

	Experiment 1	Experiment 2 (additions only)	Combined
**All Labels**	293	137	430
**Natural Labels**	140	23	163
**Artificial Labels**	120	102	222
**Ambiguous Labels**	33	12	45

### Conclusions

CSN correlates with PN it also differentiated between known restorative and nonrestorative images (high-natural and low-natural) used in [4]. Research objective 1 has been met. It is possible to predict the PN of images by listing their contents using automated image classification techniques.

## Experiment 2: Calculated Semantic Naturalness of Pseudo-Randomly Sampled Images of an Urban Area

### Material

The Google Vision API and Google Street View Image API as described above.

### Method

Sample points were generated using the QGIS version 2.8.2. Firstly a polygon was created covering the city of Edinburgh, the city bypass forming the Southern boundary, the River Almond in the Northwestern, River Esk in the East and the coast to the North. The sampling area extended approximated 200m into the sea so as to include the possibility of sampling sea views. The total area was 133 km^2^. Within this area the Generated Regular Points tool was used with point spacing of 0.005 decimal degrees and a random offset applied to each point to give a pseudo-random spread of 768 sample points with an approximate even density across the city.

For each sample point a web service was called to find the nearest Google Street View panorama. The average distance from sample point to panorama was 68m (maximum 786, standard deviation 101). The bearing from each panorama to its sample point was calculated. The Google Street Map Image API was called to extract a static image 640 by 640 pixels with an angle of view of 90° from the panorama with the virtual camera facing towards the sample point as if someone were stood at the panorama location looking towards the sample point. The image was stored for analysis.

When images had been obtained for all sample points the same procedure was used to generate labels for these images from the Google Vision API as in Experiment 1. The additional labels generated were tagged as natural, ambiguous or artificial in the same way. CSN was also derived as in Experiment 1.

It was assumed that because CSN correlated with PN in the first experiment the measurement of CSN across the 768 points in this experiment was an equivalent for measuring PN. To test this assumption a subsample of 100 representative images was taken by pseudo-randomly sampling 20 images from each quintile of CSN. These images were then scored by a panel in the same way Berman *et al* had produced PN. Images were presented individually with a choice of seven buttons ranging from 1 (not-natural) to 7 (natural). Clicking the button scored the image and advanced to the next one. The images were presented randomly so each panel member got them in a different order. The panel was composed of members of staff and associates from Royal Botanic Garden Edinburgh (n = 15 participants, average age 48, female 10). Subjects were informed of the full nature of the experiment once they had completed the evaluation and gave their written permission for their data to be used. The average of the panel score for each image was taken as the PN for that image.

As an exploratory aside the geospatial coordinates of the sample points were reverse geocoded to postal codes. These were then used to associate sample points with data zones in the Scottish Index of Multiple Deprivation (SIMD)[13]. The sample points, panorama locations, level of CSN and level of deprivation were visualised on maps using QGIS.

The images obtained from street view and sample point locations were not reviewed by the author until after other analyses were complete at which point subjective observations were made of the success or otherwise of the image classification algorithms.

### Results

A total of 299 labels were returned for the 768 sampled images. 162 of these were shared with those from Experiment 1. 137 were added new to the image pool. (Table 1 and Table 2 for summaries).

**Table 2:** Most Frequent labels from experiments 1 & 2 combined. Full list available in additional materials.

Semantic Class	Label (number of images)
Natural	tree (172), yard (123), cloud (112), garden (108), backyard (89), plant (88), flower (85), grass (84), waterway (80), habitat (77), shrub (69), sea (60), natural environment (58), lawn (46), plain (46), coast (43), forest (42), green (38), woodland (30), shore (29)
Artificial	residential area (395), property (310), vehicle (222), estate (215), suburb (204), town (166), house (162), road (145), condominium (136), automobile (133), asphalt (107), facade (78), walkway (76), home (74), flooring (71), cottage (67), hacienda (67), transport (63), driveway (63), real estate (62)
Ambiguous	area (244), neighbourhood (52), horizon (35), lane (27), produce (22), path (20), outdoor structure (16), panorama (14), farm (13), blue (9), skyline (9), phenomenon (8), reflection (7), red (6), arch (5), stone wall (5), yellow (4), resort (4), vacation (3), circle (3)

The majority of images, 584 (76%), had a CSN of less than 0 (more artificial than natural), 152 (20%) had a CSN greater than 0 and 32 (4%) were scored 0.

For the one hundred images evaluated for PN the panel returned a flattened normal distribution around the mid value of 4 shifted somewhat to artificial (sd = 1.27, kurtosis = 2.38, skewness = 0.10). The agreement between the panelists for each image was moderate with average standard deviation across the images of 1.15.

**Fig 2:**
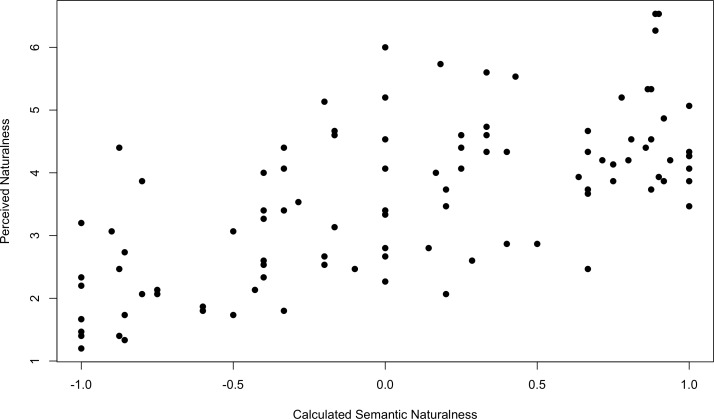
Correlation of Perceived Naturalness as scored by panel and Calculated Semantic Naturalness (Experiment 2).

Fig 2 is a plot of PN against CSN for the one hundred images presented to the panel.

Pearson's product-moment correlation of PN to CSN is 0.6659 (p<0.01) 95% confidence between 0.5401 and 0.7626—a moderately strong correlation.

756 of the 768 could be reverse geocoded and linked to SIMD data zones. There was no correlation between CSN and the SIMD 2012 vigintiles of deprivation (r = -0.0416, p = 0.2535)

**Fig 3:**
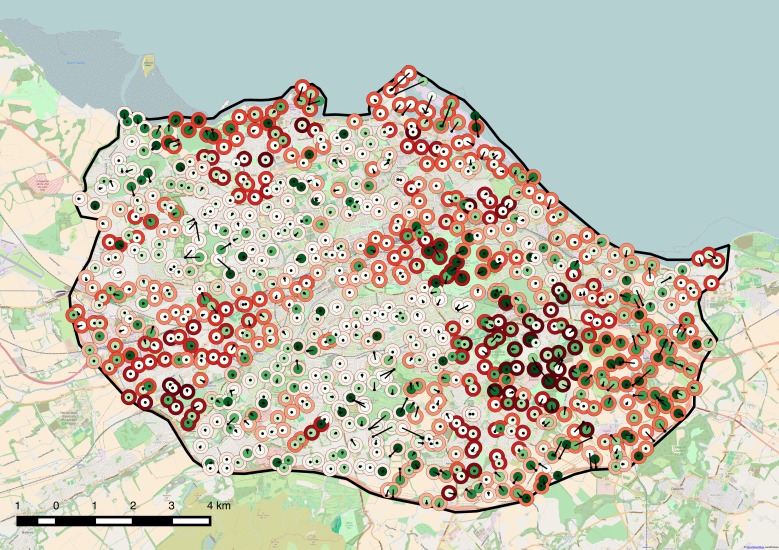
Map of Edinburgh showing sample area (Experiment 2). Greener dots are more natural. Redder halos are more deprived. Where Street View panoramas are offset they are joined to sample points by lines.

Fig 3 is a map of sample points (larger dots, greener for higher CSN), panorama locations (smaller black dots joined to associated sample point by line) and SIMD 2012 as halo around the sample point dots with areas of higher deprivation darker red.

Subjective assessment of the Street View images and sample points suggested that the number of natural subjects missed by the vision API was high and the number of false positives low. There were images where roadside specimen trees were clearly dominant but no natural labels returned. In a few cases two sample points shared the same panorama but had slightly different images extracted from that panorama resulting in distinctly different labels returned.

### Conclusion

CSN correlates with PN in randomly sampled images from an urban setting. Assessment of results suggests that as image categorisation improves, the correlation between CSN and PN will also improve. In this purely exploratory example CSN was not found to correlate with regions of deprivation but, as discussed below, this is not a rigorous test.

Research objective 2 has been met. It is possible to deploy this approach to sample data across a city.

## Discussion

This study differs from previous studies in two key aspects, it uses the semantic classification of auto detected objects in images and it uses randomly sampled, ground level images to assess naturalness rather than photographs composed by a human. It is believed that both these are novel in the study of perception of natural and potentially restorative environments. As far as possible the detection and assessment process was treated as a “black box” as the intention was to evaluate the potential for automated assessment, possibly on a large scale.

The only human input was in the assignment of labels to natural, artificial or ambiguous classes. These semantic classes were so broad that it was a very straightforward process with little ambiguity.

Semantics were an issue with the panelists scoring the Street View images for PN. They were mainly professional biologists but unfamiliar with work on restorative landscapes. When the test was explained to the first two they had no comprehension of what was meant by “natural” in this context, there being no truly natural landscapes in Scotland, their response would have been to score all images as 1. When the term was changed to “nature” they understood in the common sense of the word discussed by Kaplan and Kaplan [9].

Considering the methodology was largely plugging together commercially available components the results were remarkable robust. The participants in the two studies that produce the images and scorings for Experiment 1 were North American students in their late teens or early twenties and the composed images were of North American urban or natural scenes. This study used a panel of subjects averaging twice that age and random images of an historic European city yet both seem to indicate similar effects.

It demonstrates that, at least for this set of images, the semantics of the contents carry a similar signal of naturalness to the low level visual features assessed by Berman *et al*. The relative importance of low level features and semantic meaning were not examined although two are obviously interdependent.

With the area of artificial intelligence (AI) in rapid development and access to increased amounts of computer power through cloud based services it is likely that our ability to analyse the contents of automatically sampled images for naturalness and potentially restorative value will become a useful tool in assessment of urban areas. This study is restricted to simple, generic image classification but other analyses will likely be available that will encompass different aspects of restorative environments proposed by the major theories.

ART proposes four properties of restorative environments[9]: extent, being away, soft fascination and compatibility. SRT proposes six properties of restorative environments[5] that overlap somewhat with ART; complexity, focal point, depth, ground conducive to movement, deflected vista, low level of threat. The technique used here only examines the soft fascination and complexity properties but by enumerating objects and comparing them with lists of objects from subjects preferred environments it could go some way to addressing ART’s compatibility property. Analysis of whole panoramas rather than single images would provide an estimate of extent. Going beyond the techniques used in this study methods of measuring focal point, depth, ground conducive to movement and deflected vista are all domains required by current autonomous vehicle technology. Rangefinder measurements, from which some of these may be derived, are already recorded as part of the Google Street View survey process. The Google Vision API currently offers image classification by emotional facial attributes returning an estimate of whether faces in an images are happy or sad. In the longer term it seems feasible that a similar service could be created to return whether a place is restorative or not.

In the short term, application and enhancements of the technique used here might be informative in joining the gap between remote sensing studies [14,15], studies of individual subjects in the field [16] or lab [8]. Possible applications include looking at routes taken to work and school or the relationship between objects in the environment versus those consciously perceived.

When looking at urban green space indicators for epidemiological studies in four UK cities, including Edinburgh, Mitchell *et al* [15] concluded: “Larger green spaces may be the most important for health effects, but may also be less prevalent in more deprived areas.” A pilot study also showed that more green space is associated with lower levels of stress in deprived communities[17] in a neighbouring Scottish city. These studies seem counter intuitive for Edinburgh. Casual observation suggests that several of the areas of deprivation are associated with large areas of green space so the exploratory aside, to look at correlation with SIMD, was not expected to find a strong correlation. In the event no correlation at all was found. Significant conclusions should not be drawn from this but it does show the feasibility of applying the techniques used here in studies that examine greenspace quality, exposure and engagement for different socio economic groups.

## Overall Conclusions

Numerous studies have shown that natural environments and images of natural environments have similar restorative effects[1]. Images used in these studies have tended to be composed by humans. Berman *et al* [4] have shown a correlation between PN and restorative value as well as low level image features, PN and restorative value. This study introduced CSN and showed it correlates with PN in composed images. It went further to show that PN and CSN correlate in pseudo randomly sampled images in a city scale geospatial project but in an exploratory aside CSN was not predictive of areas of deprivation. In common with other studies, what is not shown is a direct link to locations that are restorative. All the correlations are at one remove i.e. images of locations. Providing this direct link will have to be the topic of other studies. Neither does this study provide evidence of causation. It does however establish the principle that automated sampling and analysis of ground level image data may be useful in exploring perceived naturalness and therefore possible restorative value of urban spaces.

## Supporting Information

S1 File Data Matrix Full dataset suitable for loading directly into R. (CSV)

S2 File Sample Points Geospatial details of the Edinburgh sample points. (CSV)

S3 File Street View URIs Uniform Resource Identifiers to download Google StreetView images used in the study. (TXT)

S4 File Google Labels The 430 labels returned by the Google Vision API and their scoring to natural, artificial or ambiguous. (CSV)

S5 File Column Legends Details of columns in S1, S2 and S3 Files. (TXT)
